# Diagnostic value of 3T whole heart coronary magnetic resonance angiography (MRA) without contrast medium

**DOI:** 10.1186/1532-429X-15-S1-E61

**Published:** 2013-01-30

**Authors:** Ryuzo Nawada, Natsuko Hosoya, Shigetaka Kageyama, Toru Yoshizaki, Atsushi Sakamoto, Ryosuke Takeuchi, Koichiro Murata, Tomoya Onodera, Akinori Takizawa

**Affiliations:** 1 Cardiology, Shizuoka City Shizuoka Hospital, Shizuoka, Japan

## Background

Until quite recently, 3T whole heart coronary MRA needed contrast medium to get adequate image quality. Lately, 3T MRI scanner with novel technology made possible to get good image quality without contrast medium. We evaluated the diagnostic value of 3T whole heart coronary MRA without contrast medium.

## Methods

From May 1 to September 6 in 2012, 31 consecutive patients received whole heart coronary MRA without contrast medium in 3T MRI scanner (Ingenia 3.0T, Philips Healthcare). 26 patients (84%) had sufficient quality to analyze. In these, 21 patients (16 men; mean age 69 years) received coronary angiography within 1 month of MRA. In MRA, coronary arteries were segmented to proximal, mid, distal RCA (segment 1, 2, 3), LMT (segment 5), proximal, mid, distal LAD (segment 6, 7, 8), and proximal, distal LCX (segment 11, 13). In coronary angiography, a stenosis of over 50% was defined to be significant. The segments after stent implantation were excluded.

## Results

A total of 175 segments were analyzed. Sensitivity, specificity, accuracy, positive predictive value (PPV) and negative predictive value (NPV) in whole heart coronary MRA were 83%, 90%, 89%, 63% and 96%, respectively. Furthermore, these indexes were 82%, 93%, 91%, 75% and 95% in RCA, 80%, 84%, 83%, 63% and 93% in LAD, 100%, 89%, 90%, 43% and 100% in LCX. In LMT, there were no significant stenosis in all subjects, and specificity and NPV were both 100%.

## Conclusions

In our first experience, 3T whole heart coronary MRA without contrast medium had high specificity and NPV, but sensitivity and PPV were not satisfactory. There were relatively high PPV in RCA.

## Funding

None.

